# Medication costs during an 18 month clinical trial of obesity treatment among patients encountered in primary care

**DOI:** 10.1186/s40608-015-0054-4

**Published:** 2015-05-29

**Authors:** Adam G. Tsai, Elizabeth Juarez-Colunga, Sue Felton, Rebecca B. Speer, Daniel H. Bessesen, Adam J. Atherly

**Affiliations:** Division of General Internal Medicine, University of Colorado School of Medicine, Aurora, USA; Anschutz Health and Wellness Center, University of Colorado School of Medicine, Aurora, USA; Department of Biostatistics, Colorado School of Public Health, Aurora, USA; Department of Medicine, Denver Health Medical Center, Denver, USA; Department of Health Systems, Management, and Policy, Colorado School of Public Health, Aurora, USA; Kaiser Permanente of Colorado, Departments of Internal Medicine and Metabolic-Surgical Weight Management, 2045 Franklin Street, 3rd Floor, Denver, USA

**Keywords:** Obesity, Health care utilization, Medications, Clinical trial

## Abstract

**Background:**

Weight loss often leads to reductions in medication costs, particularly for weight-related conditions. We aimed to evaluate changes in medication costs from an 18 month study of weight loss among patients recruited from primary care.

**Methods:**

Study participants (n = 79, average age = 56.3; 75.7 % female) with average BMI of 39.5 kg/m^2^, plus one co-morbid condition of either diabetes/pre-diabetes, hypertension, abnormal cholesterol, or sleep apnea, were recruited from 2 internal medicine practices. All participants received intensive behavioral and dietary treatment during months 0–6, including subsidized access to portion-controlled foods for weight loss. From months 7–18, all participants were offered continued access to subsidized foods, and half of participants were randomly assigned to continue in-person visits (“Intensified Maintenance”), while the other half received materials by mail or e-mail (“Standard Maintenance”). Medication costs were evaluated at months 0, 6, and 18.

**Results:**

Participants assigned to Intensified Maintenance maintained nearly all their lost weight, whereas those assigned to Standard Maintenance regained weight. However, no significant differences in medication costs were observed within or between groups during the 18 months of the trial. A reduction of nearly $30 per month (12.9 %) was observed among all participants from month 0 to month 6 (active weight loss phase), but this difference did not reach statistical significance.

**Conclusions:**

A behavioral intervention that led to clinically significant weight loss did not lead to statistically significant reductions in medication costs. Substantial variability in medication costs and lack of a systematic approach by the study team to reduce medications may explain the lack of effect.

**Trial registration:**

The trial was registered at (NCT01220089) on September 23, 2010.

## Background

Obesity is arguably the greatest public health challenge of the early 21^st^ century in the U.S. and other developed nations. Health care payers, both public and private, are appropriately concerned about the large health care costs of obesity [1, 2]. It is often assumed that reductions in obesity will be followed by reductions in health care utilization, but this has not always been the case. For example, in the Swedish Obese Subjects (SOS) study, individuals who lost 15–25 % of their starting weight after bariatric surgery had similar medication costs after 6 years of follow-up, compared to a control group treated with diet and exercise and who lost a minimal amount of weight [3]. Similarly, an analysis of patients before and one year after weight loss surgery found no reduction in the use of opioids for chronic pain, despite an average reduction of 13 BMI units one year after surgery [4]. In fact, an increase of approximately 10 % in opiate use was observed.

Whether cost reductions are observed among individuals losing weight may depend upon the type of intervention that is used, as well as the characteristics of the intervention participants and the degree of weight loss achieved. Although weight loss surgery leads to dramatic weight reduction, the high initial costs may take years to recoup. At least two studies have demonstrated that medically supervised low-calorie diets lead to substantial short term reductions in medication costs, particularly for individuals who had co-morbid conditions when they entered treatment [5, 6]. The cost of a medically supervised low-calorie diet program is substantially less than the cost of surgery (at the current institution of the first author, a difference of approximately 7 fold). Lesser but statistically significant reductions in medication use have been observed in at least two clinical trials of weight loss *without* the use of medically supervised low-calorie diets [7, 8]. One of these two studies was Look AHEAD, in which all participants had type 2 diabetes. Smaller reductions in health care expenditures have been observed in workplace interventions [9]. It is likely that individuals with higher medication utilization at baseline (e.g., those with diabetes) will have the greatest reductions in medication use and costs following clinically significant weight loss.

Of the trials described above, only one trial was conducted among patients encountered in primary care practice [8]. Thus, this study was undertaken to address the existing gap in the literature of whether or not medication costs are reduced among primary care patients who undergo intensive lifestyle intervention for treatment of obesity. We chose specifically to study the economic outcomes of the intervention because economic outcomes of obesity treatment, in addition to clinical outcomes, have important implications for health care payers. Primary care patients were the target population because of the ongoing focus on improving the evaluation and treatment of obesity in primary care settings in the U.S. [10].

## Methods

We analyzed changes in medication costs during an 18 month clinical trial. The design, methods, and 6 month outcomes of the study have been described previously [11]. Participants (n = 79) were recruited from two primary care internal medicine practices at the University of Colorado. Participants responded after receiving a recruitment letter. They were initially screened by telephone for eligibility, and then were evaluated in-person to ensure appropriateness for participation in an extended behavioral weight loss intervention. Participants had an average age of 56.3 years, an average BMI of 39.5 kg/m^2^, and were 75.7 % female. All participants had at least one of the following co-morbidities: diabetes/pre-diabetes (34 %), hypertension (58 %), abnormal cholesterol (65 %), or sleep apnea (30 %). Participants did not have to be taking medication for the weight-related condition to be eligible. Among all participants, the average number of weight-related co-morbidities was 1.9.

All participants received intensive behavioral treatment and subsidized access to portion-controlled foods for weight loss during the first 6 months. Thus, months 0 to 6 formed the active weight loss phase of the trial. At month 6, participants were randomized to one of two treatment conditions to help them maintain weight loss: “Standard Maintenance” or “Intensified Maintenance.” All participants continued to have access to subsidized portion-controlled foods, but only those in the Intensified Maintenance group continued in-person visits during months 7–18. A total of 84 participants were randomized to a treatment condition at month 6, and of these, 79 completed 18 months of treatment; thus, the attrition rate was 6.0 %. All participants lost a clinically significant amount of weight during the first 6 months,^9^ and those assigned to Intensified Maintenance kept off significantly more weight at month 18 (data submitted for publication). The trial was approved by the Colorado Multiple Institutional Review Board (protocol #10-0719) and was registered at www.clinicaltrials.gov (NCT01220089). All participants gave informed consent prior to enrolling in the trial.

Medication names and doses were reviewed with study participants at each of the major assessment points (month 0, month 6, and month 18). Costs were assigned to each medication at the specific dose, using the ‘Big 4’ Federal Supply Schedule, the pharmaceutical price list used by the Coast Guard, the Department of Defense, the Public Health Service, and the Veterans Administration [12]. We opted not to use the average wholesale price from the “Red Book”, as U.S. government reports have suggested that these prices have been inflated by the pharmaceutical industry [13, 14].

In addition to testing changes in total medication costs, we classified medications as being used for diabetes, hypertension, or hyperlipidemia (i.e., a medication to treat a weight-related condition), or as being used for another condition. We hypothesized that if medication reductions were observed, they would most likely be seen in medications used to treat weight-related conditions. We considered classification of any medication used to treat a weight-related condition, but this was not a clear separation (e.g., non-steroidal anti-inflammatory medications and anti-depressants could potentially be classified as being medications for weight-related conditions, or could be classified as treating non weight-related conditions). Finally, we conducted post-hoc analyses to examine whether certain subsets of participants had greater reductions in medication costs. Specifically, we examined individuals with at least a 10 % weight loss, as well as those with diabetes or hypertension, who were likely to be taking the largest number of medications at baseline.

The primary outcome was cost and the main explanatory variable was treatment group. We log-transformed cost and used linear mixed models with random intercept to account for the repeated outcome at three time points (month 0, month 6, and month 18). Time was used as an explanatory variable as well as an interaction between time and treatment group to allow for differential effect of the treatment group at each time point. The smearing approach for back transforming the mean in the log scale was used to obtain an estimate of the mean cost in the original scale [15–17]. To obtain standard errors of the mean in the original scale, the bootstrap method with 2000 samples (with replacement) was used. As the distribution of the differences of cost in the original scale seemed fairly symmetric, p values were obtained assuming a normal distribution for the empirical distribution of the mean estimate. Results based on the linear mixed model were also obtained for cost of medications non-weight-related as well as weight-related. All analyses were conducted in the statistical program R [18]. We present results below for re-transformed data, in order to compare them to other published studies on this topic. Analyses using median data showed similar results. Costs are presented in 2013 U.S. dollar amounts.

## Results

All participants lost a clinically significant amount of weight during months 0–6. During months 7–18, the Standard Maintenance group regained significantly more weight than the Intensified Maintenance group (data submitted for publication). Among the 84 individuals who were randomized at month 6, 79 completed the 18 month assessment. Thus, the attrition rate was 6.0 %. No differences in age, gender, BMI, or medication costs at time of randomization were observed between the 79 individuals who completed the trial, as compared to the 5 who dropped out.

Of the 79 participants, 10 (12.7 %) took no medications at baseline. At month 0, the average number of medications per person was 3.49 ± 2.67, of which 1.68 ± 1.79 were for weight-related conditions, and 1.81 ± 1.69 were for other conditions. At month 6, the average number of medications was 3.53 ± 2.65, of which 1.71 ± 1.83 were for weight-related conditions and 1.82 ± 1.69 were for other conditions, respectively. At month 18, the average number of medications was 3.64 ± 2.72, of which 1.63 ± 1.73 were for weight-related conditions and 2.01 ± 1.99 were for other conditions.

The overall results demonstrated no significant changes in medication costs throughout the course of the trial. During months 0–6 (the active weight loss phase), mean medication costs among all study participants were reduced from $229.08 to $199.54, a decrease of 12.9 %, but this reduction did not reach statistical significance (p = 0.34). During months 7–18 (the randomized phase), there were no significant differences between the standard and intensified maintenance groups in medication costs, either in total medication costs or whether medications were separated into weight-related or non-weight-related categories (Table 1). Finally, post-hoc analyses of participants with diabetes or hypertension and those with at least a 10 % loss of initial weight showed no statistically different differences (Table 2).

**Table 1:** Differences Between Groups in Monthly Medication Costs

	Standard Maintenance	Intensified Maintenance	Between-group difference	p value
Month 0	266.83(79.11)	273.12(76.05)	6.29(115.47)	n/a^†^
Month 6	228.56(70.48)	241.84(66.7)	13.27(101.33)	0.45
Month 18	222.19(69.99)	275.29(78.35)	53.11(109.62)	0.31
Change: Month 6- Month 18				
Total cost	−6.38(104.74)	33.46(104.68)	39.83(147.52)	0.61
Weight-related medications^a, b^	−3.58(65.68)	−63.36(56.01)	59.78(86.5)	0.24
Non-weight-related medications^a, b^	−7.49(54.22)	54.32(67.7)	61.81(86.87)	0.76
Change: Month 0 – Month 18				
Total cost	−44.64(111.98)	2.18(113.8)	46.82(160.76)	n/a^†^
Weight-related medications^a, b^	−0.72(65.05)	−11.51(47.14)	10.79(81.08)	n/a^†^
Non-weight-related medications^a, b^	−31.06(61.04)	−4.37(80.72)	26.69(102.87)	n/a^†^

^a^ Data are presented as mean (se) in 2013 U.S. dollar amounts

^a, b^ Medications for weight-related conditions are those for diabetes, hypertension, and cholesterol

† P values for Month 0 and change from Month 0 to Month 18 are not applicable since these were not a priori planned comparisons (i.e., randomization occurred at month 6)

**Table 2:** Changes in Medication Costs for Subgroups^a^

	Month 0	Month 6	Month 18	Change: Month 0 to Month 6; p value	Change: Month 6 to Month 18; p value	Change: Month 0 to Month 18; p value
Diabetes (DM)	281.56	298.01	250.66	16.45	−47.36	−30.9
or Hypertension	(59.89)	(66.34)	(56.42)	(95.95);	(97.21);	(91.27);
(HTN) (n = 59)				0.57	0.31	0.37
10 % weight	232.78	215.38	213.48	−17.4	−1.9	−19.3
loss^a, b^	(102.17)	(92.43)	(94.8)	(150.74);	(148.22);	(160.1);
(n = 21)				0.45	0.49	0.45
DM or HTN and	256.05	235.86	233.43	−20.2	−2.43	−22.63
10 % weight	(102.85)	(93.18)	(96.77)	(151.39);	(152.07);	(161.71);
loss^a, b^ (n = 19)				0.45	0.49	0.44

^a^ Data are presented as mean (se) in 2013 U.S. dollar amounts

^a, b^ Participants who lost at least 10 % of starting weight from month 0 to month 6

## Discussion

In this analysis of medication cost changes from an 18 month clinical trial, we observed no significant changes in medication costs, neither during the period of active weight loss, nor between the two treatment groups for weight loss maintenance after randomization. Subgroup analyses looking at participants who lost at least 10 % of baseline weight and participants with diabetes or hypertension also did not show significant differences, possibly related to small sample size.

At least three reasons might explain why no differences between groups were observed. First, the design of the current study sought to prevent weight regain, which limited our ability to detect differences between the two study arms after randomization. Indeed, the difference between the two groups in weight at 18 months was 3.7 kg, substantially smaller than the 8.1 % difference between treatment and control groups in starting weight (approximately 8 kg) seen after 1 year in the Look AHEAD trial [19]. Second, nearly 13 % of participants took no medications at all, limiting our ability to detect differences between groups, as most of these individuals had no change in medication utilization throughout the trial. [A post-hoc power calculation suggested that, with the effect size and variance seen in the current trial, a sample size of over 2,000 participants would have been required to observe statistically significant effects]. Third, unlike the Look AHEAD Trial, where study physicians actively adjusted diabetes medications to prevent hypoglycemia as participants lost weight, we left all medication changes to participants’ personal physicians. We believe this third limitation to be the most important, given the existing evidence that obesity is not optimally managed in brief primary care visits.

Although results from the current analysis were not statistically significant, the reduction of $29.54 per month in total medication costs during the active weight loss phase (months 0–6) was similar in magnitude compared to other studies. Redmon et al reported a difference of $28 per month in medication costs from the Look AHEAD Trial (n = 4,358, all with type 2 diabetes) between the treatment and control groups after 1 year (p < 0.001) [7]. Tsai et al reported a difference in medication costs over two years in the POWER-UP Trial (n = 390, BMI of 30–49.9 kg/m^2^ and one other risk factor for cardiovascular disease) of $290 between treatment and control groups (p < 0.05), which approximated to $12 per month [8]. Collins and Anderson reported an annual savings of $443 in medication costs (n = 32, all taking medication for diabetes or hypertension) after completion of a very-low-calorie diet (i.e., approximately $37 per month) [5]. Anderson and Jhaveri reported a reduction of $100 per month in medication costs (n =183, including n = 100 with severe obesity, and all taking medications for obesity-related conditions) after a very-low-calorie diet, but only measured medication usage for 20 weeks and did not follow patients after completion of the program [6]. Wolf et al reported that a RD intervention (n = 147, BMI ≥30 kg/m^2^ and with type 2 diabetes) reduced inpatient costs substantially but did not reduce medication costs [20].

Despite non-significant results, we believe that the lack of cost differences in the current trial should not present a barrier to implementation of obesity treatment in primary care. Health care payers often expect treatment of chronic diseases to be cost saving, but the reality is that very few interventions in health care actually achieve this goal [21]. A better metric of cost-effectiveness is cost per quality adjusted life year (QALY), which can be compared across all kind of health care interventions. Full cost-effectiveness analysis was beyond the scope of our resources in the current trial, but has been conducted for other obesity treatment interventions, including the Diabetes Prevention Program [22–30].

## Conclusions

In summary, in this 18 month clinical trial, we found no significant changes in medication costs during weight loss or weight loss maintenance. We believe that the population in this trial was reasonably representative of patients encountered in primary care. However, the disparate results of this trial and of another economic analysis of a primary care-based intervention [8] suggest the need for more research in this area. The cost reductions seen during the first 6 months are consistent with previous literature, and thus, we believe these data are generalisable. However, the lack of significance in the overall results may not be generalisable to the broader population of primary care patients, given that a similar magnitude of cost reduction resulted in statistical significance in previous trials with larger samples. Future studies should continue to evaluate the economic benefits of weight reduction. In doing so, studies should attempt to bridge the gap between research and clinical practice and should help patients achieve weight loss while enhancing quality and coordination of medical care.
